# A Study of Grain Selection in Two-Dimensional (2D) Grain Selectors during the Investment Casting of Single-Crystal Superalloy

**DOI:** 10.3390/ma16114112

**Published:** 2023-05-31

**Authors:** Xintao Zhu, Fu Wang, Dexin Ma

**Affiliations:** 1 Foundry Institute, RWTH Aachen University, 52072 Aachen, Germany; 2 State Key Laboratory for Manufacturing System Engineering, School of Mechanical Engineering, Xi’an Jiaotong University, Xi’an 710049, China

**Keywords:** undercooling, 2D grain selector, single crystal, directional solidification

## Abstract

In this study, a series of Bridgman casting experiments were conducted to study the physical processes occurring in 2D grain selectors with different geometric parameters. The corresponding effects of the geometric parameters on grain selection were quantified by using an optical microscopy (OM) and a scanning electronic microscopy (SEM) equipped with electron backscatter diffraction (EBSD) function. Based on the results, the influences of the geometric parameters of the grain selectors are discussed, and an underlying mechanism accounting for the experimental results is proposed. The critical nucleation undercooling in the 2D grain selectors during grain selection was also analyzed.

## 1. Introduction

Ni-based single-crystal (SX) superalloys are widely used to produce turbine blades due to their excellent high-temperature performance [1,2]. Generally, SX blades are cast using the directional solidification process through a seeding or a selector method. In the seeding method, a seed made from the same material as the single crystal that will be prepared is placed at the bottom of the shell before pouring superheating molten metal in it. At the same time, the temperature gradient and the drawing rate are carefully controlled to obtain the single crystal, which orientation is consistent with the seed [3]. Once the liquid metal has been poured into the mold, the seed is partially melted, and the crystal will subsequently grow along the same crystallographic direction as the seed. If no stray crystals formed, a single crystal will be obtained. It should be noted that the seed material must have the same composition as the blade alloy [4,5,6].

The grain selection method is the most extensively used process for preparing Ni-based single-crystal superalloy turbine blades [7,8]. In the grain selection method, a grain selector is placed at the bottom of a casting or a blade, and a directional solidification technique is used to control the temperature gradient of the solid–liquid interface to obtain a single crystal [9]. Normally, the grain selector consists of two parts: a starter block and a selector part. During unidirectional solidification, solidification begins with a random nucleation of equiaxed grains on the surface of a chilled plate. Then, the equiaxed grains gradually transform toward a directional structure with the progressing of solidification. For face-centered cubic (FCC) Ni-based superalloys, the <001> direction is the preferred growth direction. Therefore, better <001>-oriented structures will form at the top of the starter block. Then, through the grain competitive growth in the selector part, one grain would finally be selected and fill the full mold cavity, attaining a single-crystal cast.

Although the seeding method can control the orientation of SX structures, stray grain also forms, and the corresponding process is complex. Due to these reasons, the grain selector method is preferred in practice. A selector is broadly composed of two parts: the starter block and the selector part. The starter block controls the orientation and the grow dynamics of the grains, while the selector part favors the growth of individual grains and ultimately determines the formation of the SX structure.

Since the advent of the grain selection method, continuous explorations have been carried out to find effective geometric structures for grain selection, and a variety of geometric shapes have been designed. Higginbotham classifies the common selectors into one of four types according to their geometry: spiral (Helix), inclined, angled, and necked [9]. Zheng et al. [10] compared the grain selection behaviors of spiral and necked selectors. The results show that the spiral selector possesses both the structure and the principle of the necked and angled selectors. In a spiral selector, the superior grain selection function is obtained from the coupling effect of the crystal’s preferential lateral growth and the spiral structure. Meanwhile, in a necked selector, there is only one physical barrier to realize grain selection, and the benefits of multiple necked structures are limited [11,12,13]. Regarding the transition-type selector, stray grains often form at abrupt corners during grain selection, which affects the final single-crystal orientation. Accordingly, the turning-type selector is not used in industrial production. Dai et al. [14,15,16] conducted a study on the evolution of grain structure and the grain selection behavior of a spiral selector by means of numerical simulation and experiment, and they found that the success of spiral grain selection arises from the geometric binding effect of the spiral channel on dendrite growth. At the same time, they argued that the geometric parameters of the spiral selector do not have a significant impact on the orientation of the final single crystal. It can be seen that some controversies still remain over the grain selection behavior of selectors and the effects of selectors on the final single-crystal orientation during Ni-based single-crystal superalloy manufacturing. Hao et al. [17] supposed that, since there is no novel nucleation in the spiral channel during grain selection, the orientation of the selected single crystal has the orientation of the grain initially entered into the spiral channel. Moreover, this orientation has a certain randomness. Meanwhile, as grain selection is realized through the physical obstruction in the spiral part, the relative spatial position of grains at the inlet of the spiral channel will directly determine the development of grains. However, they did not point out which position at the entrance of the spiral channel is more likely to form the single crystal.

In contrast, no abrupt corner exists in a spiral selector; instead, the spiral is a structure that continuously climbs in a three-dimensional (3D) space at a certain angle. In the manufacturing process, the inhomogeneous nucleation problem that results from intense lateral heat dissipation from steep edges is almost eliminated in Helix [18]. At the same time, a 3D spiral channel has a good constraint on crystal grow, which increases the grain selection efficiency. Because of these features, spiral selectors have become the most widely used selector type. Meng et al. [19] studied the grain selection efficiency of different selectors in single-crystal casting under a certain selector height. The results show that as the screw angle and the spiral wire diameter increase, the portion occupied by the single-crystal structure in the spiral gradually increases. Seo et al. [20] found that lowering the casting temperature can increase the nucleation density on the surface of a water-cooling copper plate, and the yield rate of the single crystal increases correspondingly.

In short, plentiful research studies [21,22,23,24,25,26,27,28] have been carried out on the grain selection behavior of spiral grain selectors using experimental and numerical simulation methods. Yet, to date, grain selection behavior during the manufacturing process of Ni-based single-crystal superalloys is still not completely clear, especially in relation to the competitive growth of inner-dendrites in the spiral channel. Additionally, a wax injection stage is used in industrial processes, and a spiral selector cannot be fabricated integrally with the blade due to the complexity of its three-dimensional (3D) shape. It needs to be welded with the blade later, which may bring about some inevitable position errors and sudden failures in grain selection. Moreover, a high stability cannot be guaranteed in a spiral grain selector, although it is employed extensively in casting foundries. Therefore, it is necessary to further study a suitable grain selector.

To obtain high-efficiency grain selectors, two types of novel two-dimensional (2D) grain selectors, C-form and Z-form, were designed and systematically studied in this work. A 2D model has a better observability when studying a dendrite growth process, and 2D selectors are easier to use when opening metal molds for wax pattern and cost less in industrial applications.

## 2. Experimental Procedures

### 2.1. Test and Calculation Method

#### Experimental Method

A series of Bridgman casting experiments were conducted at the Foundry Institute of the RWTH Aachen University to study the physical processes occurring in the 2D grain selectors with different geometric parameters. The corresponding effects of the geometric parameters on grain selection were quantified using an optical microscopy (OM) and a scanning electronic microscopy (SEM) equipped with electron backscatter diffraction (EBSD) function. Based on the results, the influences of the geometric parameters of the grain selectors are discussed, and an underlying mechanism accounting for the experimental results is proposed.

The commercial alloy CM247LC developed by Cannon-Muskegon in conjunction with MTU and Cannon-Muskegon Corporation, respectively, was used in the present study as it is economically representative in the industry. CM247LC is a Ni-based superalloy with a high γ’ Ni3(Al, Ti) volume fraction (62%) and a high refractory element (Ta + W + Mo) content (13.7 wt.%). CM247LC is also a low-carbon, first-generation single-crystal superalloy. The chemical compositions of the CM247LC used in the experiments are listed in Table 1.

**Figure 1:**
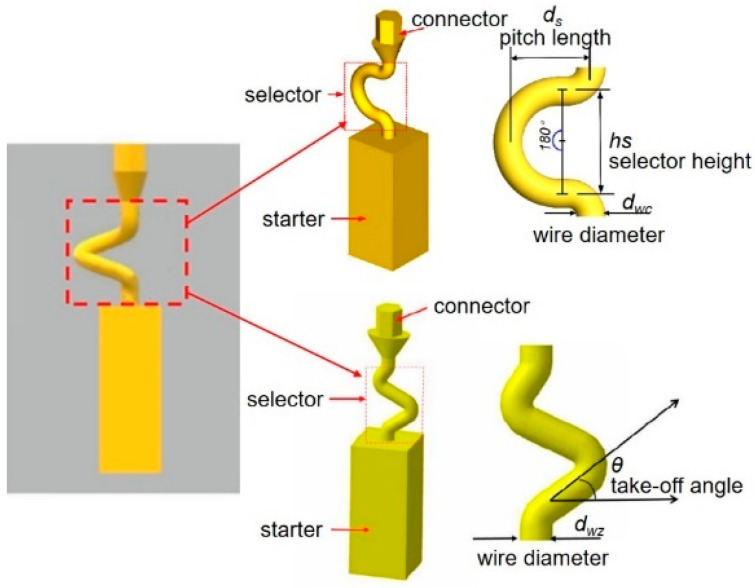
Schematic drawing of the 2D grain selectors, C-form and Z-form.

The selector part is one important portion of a grain selector and determines the ultimate grain selection result. Plenty of studies have shown that the geometric shape of a grain selector has an important influence on the selection efficiency of grains and the orientation of the final single crystal. Therefore, the key geometric parameters for the selector part of the 2D selectors, including the wire diameter, the pitch length, and the take-off angle, were systematically studied in this study to optimize the grain selector design (Figure 1). Based on the experimental results, the influences of the geometric parameters of the selectors on the grain selection efficiency are discussed, and the corresponding underlying mechanism is proposed.

In the C-form grain selectors, the selector portion was designed with varying wire diameters (2.6 mm–6.6 mm) and pitch lengths (4 mm–26 mm) under a fixed height and a curved angle (θ = 180°). Table 2 and Table 3 summarize all the C-form grain selectors, with a total of 16 cases being divided into 2 groups.

For the Z-form selectors, various take-off angles (θ = 15°–55°) and wire diameters (1.8 mm–6.0 mm) were employed. A summary of the Z-form grain selectors used in this study is presented in Table 4 and Table 5, which contain 15 cases divided into 2 groups.

The grain selector casting samples are divided into 4 tables, with numbering starting from S(sample)C(type)d(wire diameters). For example, SCd stands for the samples with C type with varying wire diameters (2.6 mm–6.6 mm).

### 2.2. Numeral Simulation

On the one hand, it is difficult to observe the evolution of solidification in real time in a common SX casting process. On the other hand, a numerical simulation can offer copious valuable information, such as the evolution of the temperature field during solidification, and it can dramatically reduce the time and experimental cost required to optimize the solidification process [26,27,28,29,30,31,32,33,34,35,36,37]. Therefore, this study adopted the software ProCAST-2017 [38] to analyze the grain selection process in the SX casting process.

To provide insight into the thermal conditions arising from experimentally varying the geometric parameters of the samples, a solidification simulation was conducted using ProCast. The temperature field and the corresponding microstructure evolution in the 2D selectors with various geometric parameters were simulated using the software ProCAST through the CAFE method.

The modular design, which is suitable for all casting processes, has the following elements:

- Finite element technology, which is capable of fully simulating thermal flow stress without the need for coupling with a third-party software.
- Accurate geometry description, which is excellent for complex shapes.
- Integrated CAD (Computer-Aided Design)/CAE (Computer-Aided Engineering), which can read data from a mainstream software directly and has a powerful geometry repair capability [38].

With the help of the finite element method, ProCAST is also capable of calculating the heat transfer (including thermal radiation and angular coefficient), the filling flow process, the thermal field-coupled stress, the microstructure, and the shrinkage.

Accordingly, the temperature field and the microstructure of the CM247LC superalloy during grain selection in various selectors were calculated using ProCAST with the actual production conditions [38,39].
**Material****Temperature (°C)**Casting1475Chill plate750Mold1550CoolantT = 400 °C for cropped Bridgman modelBaffle750Heater1550Chamber150

## 3. Results and Discussion

### 3.1. Selection Behavior and Efficiency of 2D Grain Selectors

**Figure 2:**
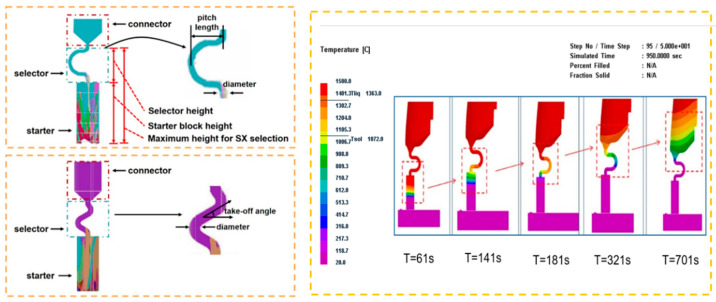
Schematic drawing of the C-type and Z-type selectors and the evolution of the temperature field in a 2D C-form grain selector.

The 2D grain selector designs (C-type and Z-type) were obtained through projecting a 3D spiral grain selector in the horizontal plane and the vertical plane, respectively. Figure 2 is a schematic drawing of the C-type and Z-type selectors, and the critical parameters in the selector part are marked in the magnified illustrations.

It can be seen that with the progression of solidification, the interval space between the isotherms gradually increases as does the width of the paste zone (Figure 2). Moreover, the shapes of the temperature field and the isotherm under different wire diameters are almost the same.

In the simulation, the starter part of the grain selectors was fixed with a size of 10 mm (L) × 10 mm (W) × 30 mm (H), while the selector portion was designed by varying the take-off angles (θ = 15°–55°) and the wire diameters (2.6 mm–6.6 mm), as shown in Table 6, Table 7 and Table 8.

**Figure 3:**
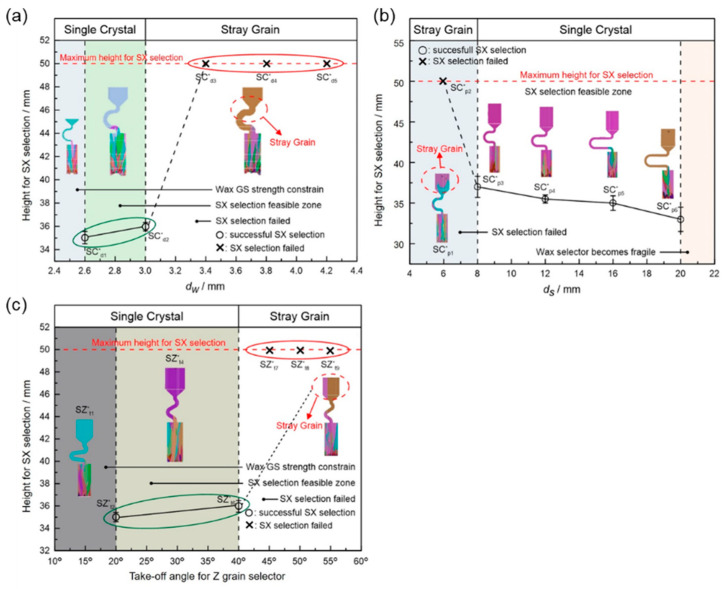
Relationship between the height for SX selection and the wire diameter in the C-form grain selector (**a**); relationship between the height for SX selection and the pitch length in the C-form grain selector (**b**); and relationship between the height for SX selection and the take-off angle in the Z-form grain selector (**c**). * means the simulation serial number.

A diameter of 3 mm is the critical value for the selector wire diameter: when the wire diameter is in the range of 2.6 mm–3 mm, a SX can be selected, as marked by the solid green circle. Figure 3a shows when the diameter is larger than SC_d2_ (3 mm), the SX selection fails, as marked by red circles. Figure 3b shows that when the pitch length is ≥8 mm, a single crystal can be selected. Otherwise, the SX selection fails, as noted below by the red circle. In Figure 3c, a single crystal can be achieved when the take-off angle is less than or equal to 40° (circled in green). Contrastingly, stray grains occur with a take-off angle larger than 40° (circled in red).

The selector part is an important portion of a grain selector and determines the ultimate grain selection result [14,15,16]. Plenty of studies have shown that the geometric shape of a grain selector has an important influence on the selection efficiency of grains and the orientation of the final single crystal [24]. Therefore, the key geometric parameters for the selector part of the 2D selectors, including the wire diameter, the pitch length, and the take-off angle, were systematically studied in this study to optimize the grain selector design. based on the experimental results, the influences of the geometric parameters of the selectors on the grain selection efficiency are discussed, and the corresponding underlying mechanism is proposed [17,18,19,20,21,22].

**Figure 4:**
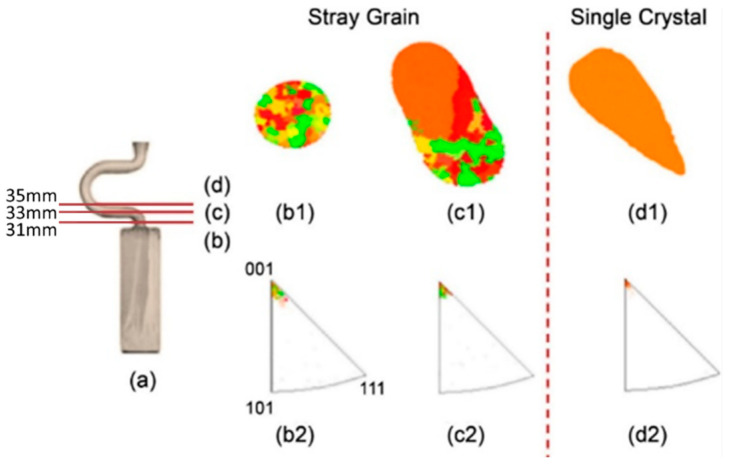
Optical image of the selector part of a C-form grain selector with a 3 mm wire diameter and a 8 mm pitch length (**a**), the EBSD maps of the grain structure (**b1**,**c1**,**d1**), and the inverse pole figures at different heights (**b2**,**c2**,**d2**).

Figure 4 shows the grain structure evolution in the selector part of a C-form selector with a wire diameter of 3 mm and a pitch length of 8 mm. It is found that with increasing height, the quantity of grains decreases significantly. Additionally, the grains also enlarge gradually (shown in Figure 4b1,c1,d1). At a height of approximately 37 mm, a single crystal is selected.

**Figure 5:**
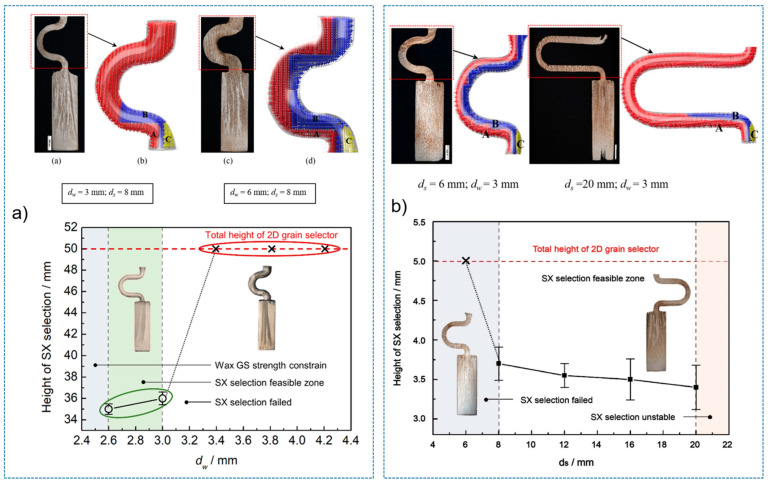
Relationship between the height for SX selection and the wire diameter (dw) of the selector (**a**), and relationship between the height for SX selection and the pitch length (ds) of the selector (**b**).

Figure 5a shows the effect of wire diameter on grain selection in a C-form grain se-lector. The pitch length is kept as 8 mm. It can be seen that as the wire diameter increases from 2.6 mm to 3 mm, a single crystal can be selected, and the height, where the SX structure is obtained, gradually increases. However, the SX selection fails when the wire diameter exceeds 3 mm. Figure 5b illustrates the effect of pitch length on grain selection. The selector wire diameter is held at a constant of 3 mm. It is found that a SX can be successfully selected when the pitch length is larger than 8 mm. Moreover, the selecting height reduces with the pitch length.

**Figure 6:**
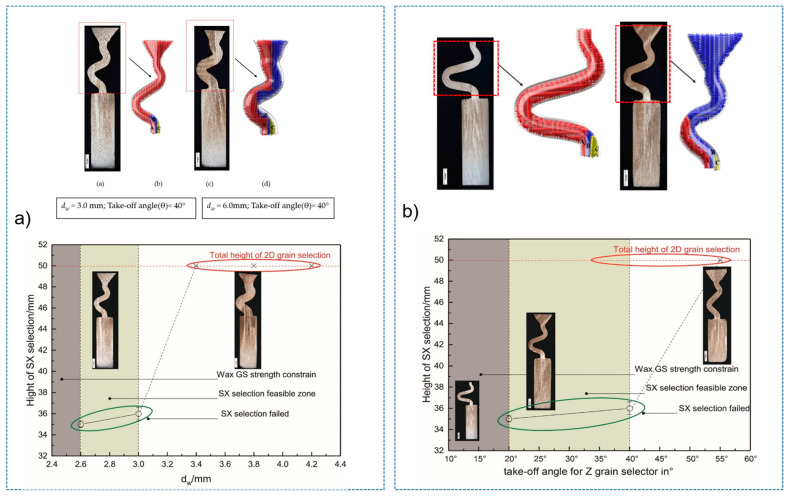
Relationship between the height for SX selection and the wire diameter (dw) of the selector (**a**), and relationship between the height for SX selection and the take-off angle of the selector portion (**b**).

The influence of wire diameter on grain selection in a Z-form grain selector is shown in Figure 6a. When the take-off angle is set to be 40° and the selector wire diameter increases from 2.6 mm to 3 mm, a single crystal can be obtained, and the height for the SX selection increases correspondingly. However, when the wire diameter is beyond 3 mm, the SX selection fails. In addition, when the wire diameter is less than 2.6 mm, the casting part is prone to break due to the low breaking strength of the selector. Figure 6b illustrates the effect of take-off angle on grain selection. The wire diameter is held constant at 3 mm. It is found that a SX can be successfully selected when the take-off angle (θ) is smaller than 40°, as higher breaks mean higher geometrical obstruction. However, when the take-off angle is smaller than 15°, structural instability occurs.

### 3.2. Influence of Undercooling on Grain Selection

Stray grain is one of the most common defects in single-crystal casting manufacturing. It breaks the integrity of the single crystal and damages the high-temperature performance of the single crystal [21,22,23,24,25,26,27,28]. Generally, stray grain is caused by undercooling, especially at corners or edge positions, where heat dissipates at a fast rate. When the undercooling is superior to the critical nucleation undercooling (ΔT_N_) of the molten metal, stray grain nucleates and grows. The critical nucleation undercooling characterizes the capability of a molten metal to keep the liquid state without nucleation in a temperature below the melting point. It is not only determined by the chemical composition but is also affected by the mould shell and the temperature evolution during solidification. Therefore, the undercooling in a 2D grain selector was analyzed in this study to help understanding the underlying grain selection mechanism.

**Figure 7:**
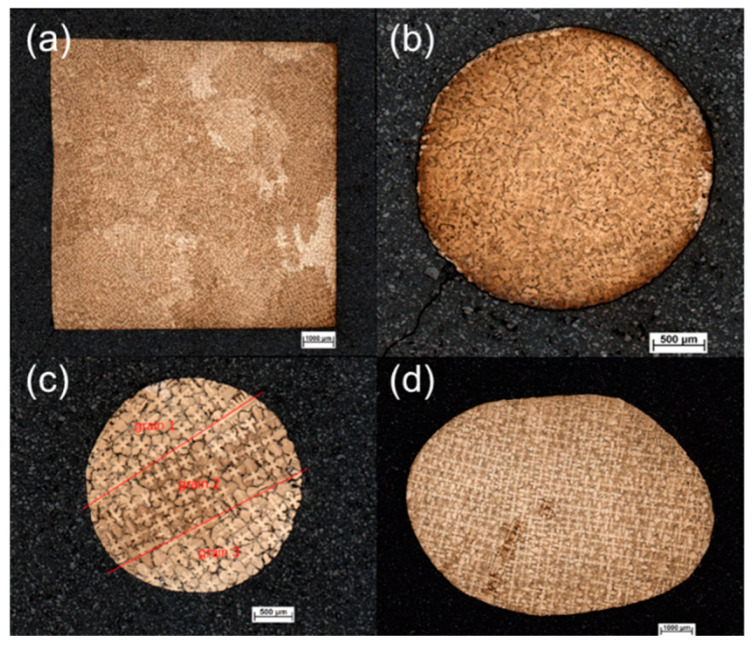
Microstructure of cross sections at heights of 25 mm (**a**), 32 mm (**b**), 40 mm (**c**), and 55 mm (**d**) in the C-form grain selector with a pitch length of 6 mm (SC_p2_).

Figure 7 shows the morphologies of the cross sections at heights of 25 mm, 32 mm, 40 mm, and 55 mm in the grain selector SC_p2_. It is clear that a lot of grains appear at the initial stage. However, the number of grains reduce drastically, especially at a height of 40 mm, as shown in Figure 7c, where 3 grains exist.

**Figure 8:**
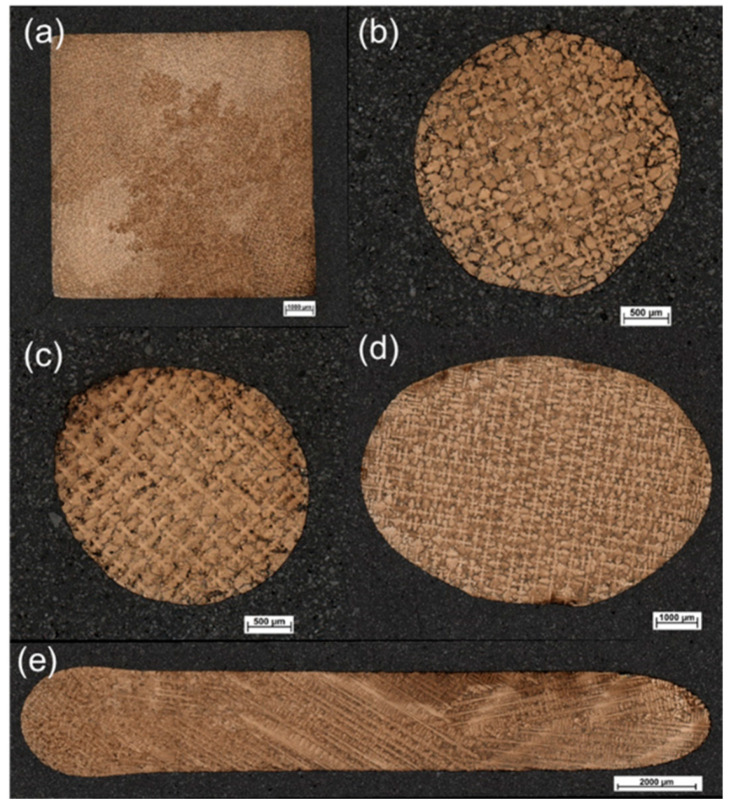
Microstructure of cross sections at heights of 25 mm (**a**), 40 mm (**b**), 48 mm (**c**), 55 mm (**d**), and 35 mm (**e**) in the grain selector with a pitch length of 8 mm (SC_p3_).

**Figure 9:**
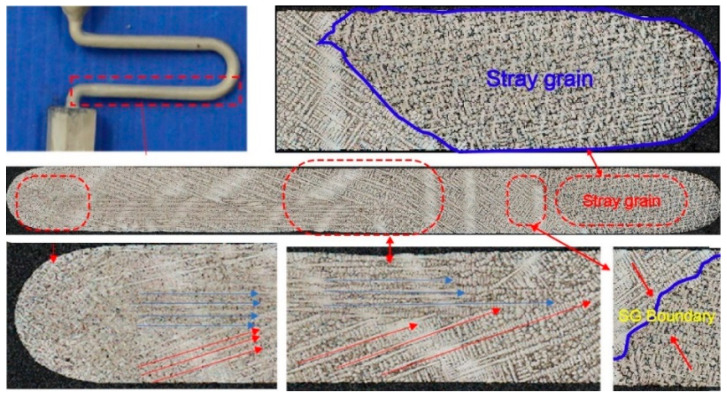
Schematic of dendrite growth at the first platform of the grain selector (SC_p8_).

Figure 8 presents the morphologies of the cross sections at heights of 25 mm, 32 mm, 40 mm, 55 mm, and 35 mm in the grain selector SC_p3_, with the result of the SX casting. Additionally, Figure 9 shows the schematic of dendrite growth at the first platform of the grain selector (SC_p8_), with the result of stray grain formation.

**Figure 10:**
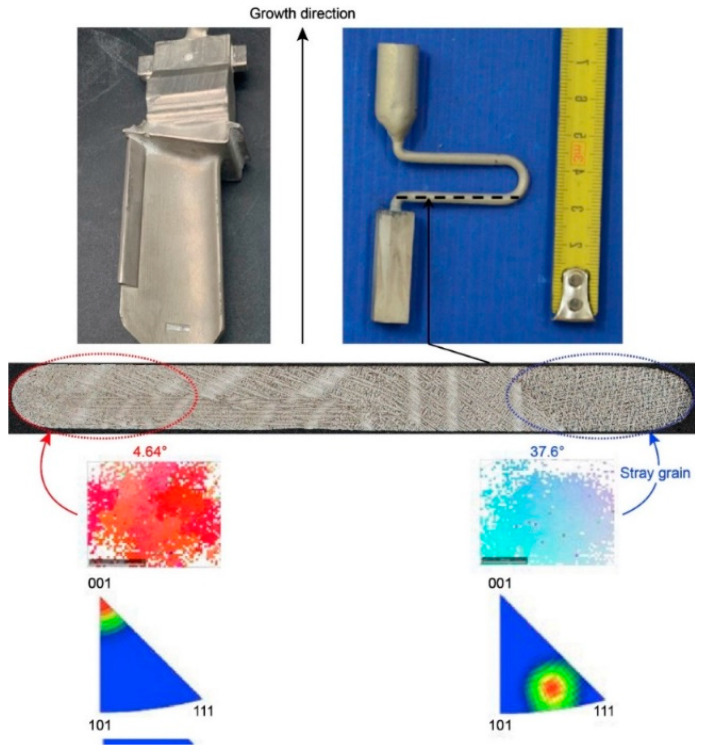
Formation of a single-crystal blade with a large deviation angle.

In Figure 10, due to the undercooling at the grain selector platform, stray grain forms and eventually evolves into a crystal with a large deviation angle.

It is important to avoid the occurrence of stray grains during single-crystal blade manufacturing. Generally, stray grains are caused by undercooling, especially at the corners or edge positions of the grain selector, where heat dissipates at a fast rate. It is affected by the mould shell and the temperature evolution during solidification. Due to the complex geometry of the grain selector, the solidification conditions at local regions are complicated, which might easily cause the formation of stray grains. According to previous research studies, stray grains may form due to the sudden change in geometry in the casting path, and Ma et al. pointed out that heat dissipation at the convex corners of the platform is so fast that undercooling forms. When the undercooling is superior to the critical nucleation undercooling (ΔT_N_) of the molten metal, stray grains nucleate and grow. The critical nucleation undercooling (ΔT_N_) characterizes the capability of a molten metal to keep the liquid state without nucleation at a temperature below the melting point.

**Figure 11:**
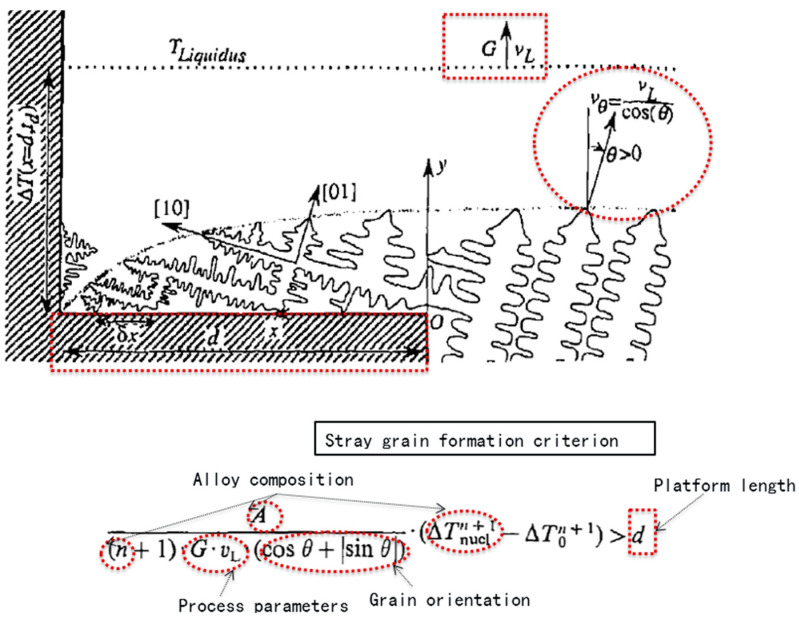
Schematic representation of dendrite growth illustrating the mechanism of competitive growth during directional solidification [40].

According to the above discussions, it could be seen that when an alloy has a low undercooling capacity, the rapid decrease in temperature at the platform corners in a selector will lead to undercooling and the formation of stray grains. From another point of view, if the undercooling ability of an alloy is large, the lateral dendrites at the primary platform of the selector might also grow at the corners and block the occurrence of stray grains, as shown in Figure 11.

Based on the heterogeneous nucleation hypothesis and the KGT model, stray grain formation at the long pitch platform of a selector can be depicted as follows:

(1) $$\frac{A}{\left(n+1)\cdot G\cdot v_{L}\cdot (cos\;\theta +\left|\sin\;\theta \right|\right)}\cdot (\Delta T_{\mathrm{nucl}}^{n+1}-\Delta T_{0}^{n+1})>d\;$$

A and n are the kinetic parameters of dendrite tip growth, which are related to the alloy composition. G is the temperature gradient; v_L_ is the growth rate; and θ is the angle between the <001> direction of the dendrite and the casting axis. ΔT_nucl_ is the critical nucleation undercooling related to the alloy composition, and d is the platform length, which is equal to the pitch length of the grain selector. ΔT_0_ is the actual undercooling at the corner of the platform.

According to the EBSD results of the SC_p2_, SC_p3_, and SC_p6_, when the pitch length is less than 20 mm, a single crystal can be selected. When the pitch length reaches 26 mm, a stray grain appears with a deviation angle of 37.6°. The actual undercooling of the molten metal at the maximum pitch length (26 mm) exceeds the critical value of 25 °C, resulting in the nucleation and growth of stray grains.

**Figure 12:**
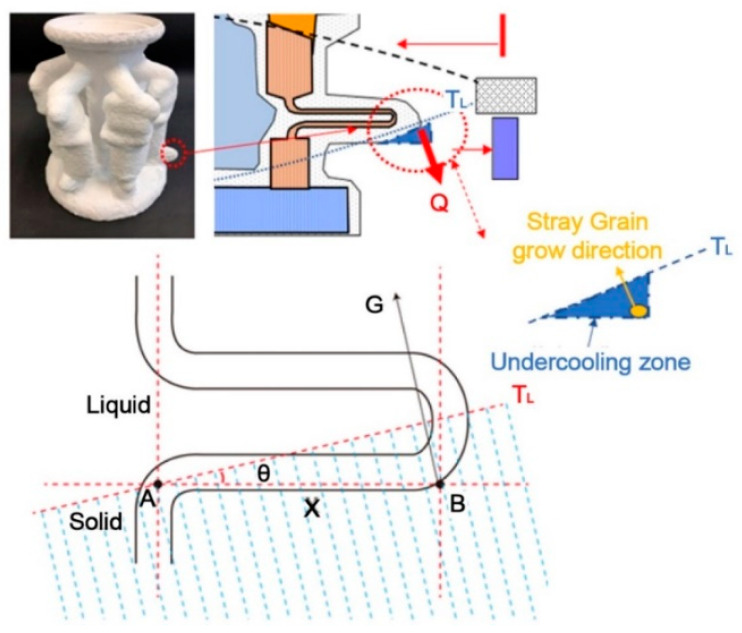
Formation of the geometry-related stray grains.

As shown in Figure 12, A and B are the start position and the end position in the first platform of the selector, and X is the transverse distance from position A. The temperature of the liquid, T_L_, in this part is set as an oblique line with an angle of θ to the horizontal plane. When the direction of the temperature gradient G is along the normal direction of T_L_ isotherm, the T_L_ isotherm is used as the solidification front temperature, while ignoring the undercooling of the dendrite growth front. Then, after T_L_ increases to the position B, an undercooling zone forms there, from the bottom to the top. When T_L_ increases to the position A, that is T_A_ = T_L_, position B obtains the highest structural undercooling:

∆T_B_ = T_L_ − T_B_ (2)

which can also be presented by the product of the transverse component of the temperature gradient G and the distance x:

∆T_B_ = G ∗ sin *θ* ∗ x (3)

If the critical nucleation undercooling of the alloy is ΔT_N_, the condition for the non-occurrence of inhomogeneity nucleation at point B would be ΔT_B_ < ΔT_N_, i.e.,

G ∗ sin *θ* ∗ x < ∆T_N_ (4)

Therefore, when the critical nucleation undercooling of the alloy CM247LC and the corresponding pitch length are determined, the pitch length criterion coefficient, J, can be calculated with the following equation:

J = ΔT_N_/ds (5)

**Figure 13:**
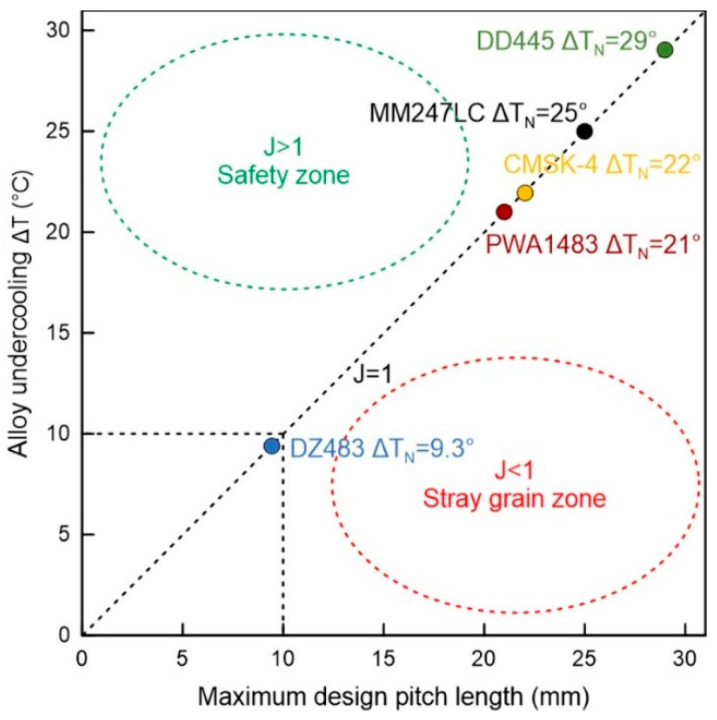
Evolution of undercooling with the designed pitch lengths.

As shown in Figure 13, when the criterion factor J is greater than one, a SX is formed, and a green safety zone appears. When the criterion factor J is less than one, stray particles form. Additionally, it is clear that the critical J value for the stray grain zone is one. The actual undercooling is directly proportional to the maximum critical pitch length when casting with a certain alloy.

Therefore, the pitch cannot be too long; otherwise, it would lead to lateral undercooling, which could also cause stray grain.

## 4. Conclusions

In the present study, the 2D grain selectors, C-form and Z-form, were designed and systematically studied to improve the grain selection efficiency of Ni-based SX castings. The following conclusions can be drawn from this study: (1) A smaller wire diameter, a smaller take-off angle, and a larger pitch length are more efficient for geometrical blocking in grain selection. A diameter of 3 mm is the critical value for the wire diameter of the selector part. For the investigated dimensions of the selector, a single crystal is achieved at the end of the selector for a diameter ≤ 3 mm, whereas for a diameter >3 mm, other dendrites also pass through the selector and stray grains are formed. For the take-off angle, the larger the value is, the smaller the deflection angle of the selected SX is. A small deflection angle can be achieved by a 2D grain selector with a take-off angle of ≤40°. The best range of pitch length is found for 8 mm–20 mm. Based on the comparison, a diameter of 3 mm, a take-off angle of 40°, and a pitch length of 8 mm are recommended for the grain selector.

(2) In the C-form selector, a smaller selector wire diameter and a larger pitch length are more efficient for grain selection. Considering the stability of the selector part, a wire diameter and a pitch length of 3 mm and 8 mm are recommended, respectively. The grain selection in the selector part during solidification is dominated by the geometrical blocking and the local thermal conditions.

(3) In the Z-form selector, the grain selection efficiency largely depends on the selector wire diameter and the take-off angle. When the selector wire diameter is smaller and the take-off angle is smaller, the whole grain selection efficiency is higher. Considering the stability and the efficiency, a selector wire diameter of 3 mm and a take-off angle of 40° are the optimal parameters for the Z-form grain selector. The coarsening phenomenon of dendrites at the turning parts is mainly caused by a rapid change in the heat flow vector, which leads the solute to diffuse slowly and accumulate at the turning areas, resulting in a decrease in the cooling rate.

(4) Undercooling plays a critical role on the formation of stray grain. Additionally, undercooling at the end of the first platform of the C-form grain selector increases with pitch length, and the tendency for stray grain formation increases. Therefore, the pitch length of the selector should be controlled in order to avoid heterogeneous nucleation.

## Figures and Tables

**Table 1:** The composition of superalloy CM247LC (wt.%).

Alloy	Elements (wt.%)									
	Al	Ti	Cr	Mo	Co	W	Ta	Hf	C	Ni
Wt.%	5.49	0.74	8.03	0.5	9.41	9.87	2.9	1.36	0.094	Bal.

**Table 2:** SC_d_-form grain selectors (C-form selector samples) with different wire diameters: Group 1.

C-Form Grain Selector with Variant Diameters	Single-Crystal		Stray Grain					
	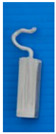	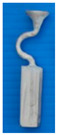	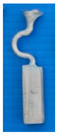	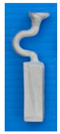	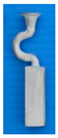	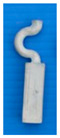	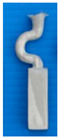	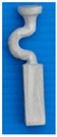
Stray grain	No		Yes					
Probe	SC_d1_	SC_d2_	SC_d3_	SC_d4_	SC_d5_	SC_d6_	SC_d7_	SC_d8_
Diameter (mm)	2.6	3.0	3.4	3.8	4.2	5.4	6.0	6.6
Pitch Length (mm)	8							
Selector Height (mm)	10							
Starter Block Size (mm)	10 (L) × 10 (W) × 30 (H)							

**Table 3:** SC_p_(pitch length)-form grain selectors (C-form selector samples) with different pitch lengths: Group 2.

C-Form Grain Selector with Variant Pitch Lengths	Stray Grain		Single Crystal					
	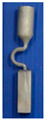	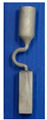	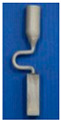	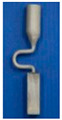	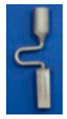	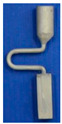	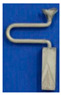	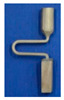
Stray grain	Yes		No					
Probe	SC_p1_	SC_p2_	SC_p3_	SC_p4_	SC_p5_	SC_p6_	SC_p7_	SC_p8_
Pitch Length (mm)	4	6	8	12	16	20	22	26
Diameter (mm)	3							
Selector Height (mm)	10							
Starter Block Size (mm)	10 (L) × 10 (W) × 30 (H)							

**Table 4:** SZ_t(take-off angle)_-form grain selectors (Z-form selector samples) with different take-off angles: Group 1.

Z-Form Grain Selector with Variant Take-Off Angles	Single Crystal						Stray Grain		
	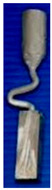	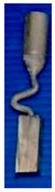	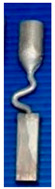	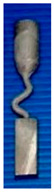	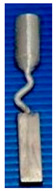	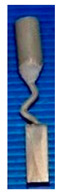	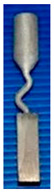	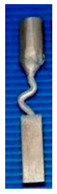	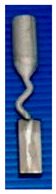
Stray grain	No						Yes		
Sample	SZ_t1_	SZ_t2_	SZ_t3_	SZ_t4_	SZ_t5_	SZ_t6_	SZ_t7_	SZ_t8_	SZ_t9_
Take-off angle	15°	20°	25°	30°	35°	40°	45°	50°	55°
Diameter (mm)	3								
Selector Height (mm)	10								
Starter Block Size (mm)	10 (L) × 10 (W) × 30 (H)								

**Table 5:** SZ_d(diameter)_-form grain selectors (Z-form selector samples) with different diameters: Group 2.

Z-Form Grain Selector with Variant Diameters	Single Crystal			Stray Grain		
	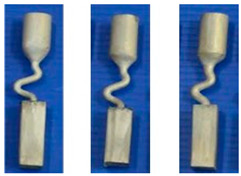			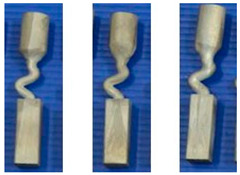		
Stray grain	No			Yes		
Probe	SZ_d1_	SZ_d2_	SZ_d3_	SZ_d4_	SZ_d5_	SZ_d6_
Diameter (mm)	1.8	2.2	3.0	4.2	5.0	6.0
Take-off angle	30°					
Selector Height (mm)	10					
Starter Block Size(mm)	10 (L) × 10 (W) × 30 (H)					

**Table 6:** Variation in wire diameter in the C-form grain selector. (SC * means the simulation sample number, in order to distinguish with experiment samples SC).

C-form Grain Selector with a Varied Wire Diameter	Single Crystal				Stray Grain			
	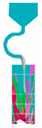	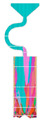	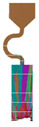	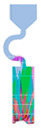	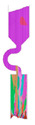	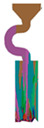	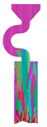	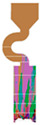
Stray Grain	No				Yes			
Probe	SC *_d1_	SC *_d2_	SC *_d3_	SC *_d4_	SC *_d5_	SC *_d6_	SC *_d7_	SC *_d8_
Diameter (mm)	2.6	3.0	3.4	3.8	4.2	5.4	6.0	6.6
Pitch Length (mm)	8							
Selector Height (mm)	10							
Starter Block Size (mm)	10 (L) × 10 (W) × 30 (H)							

* means the simulation serial number.

**Table 7:** Variation in pitch lengths in the C-form grain selector.

C-Form Grain Selector with a Varied Pitch Length	Stray Grain		Single Crystal					
	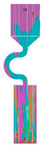	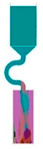	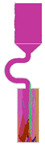	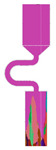	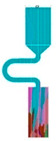	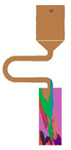	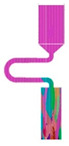	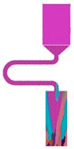
Stray Grain	Yes		No					
Probe	SC *_p1_	SC *_p2_	SC *_p3_	SC *_p4_	SC *_p5_	SC *_p6_	SC *_p7_	SC *_p8_
Pitch length (mm)	4	6	8	12	16	20	22	26
Diameter (mm)	3							
Selector Height (mm)	10							
Starter Block Size (mm)	10 (L) × 10 (W) × 30 (H)							

* means the simulation serial number.

**Table 8:** Variation in take-off angle in the Z-form grain selector.

Z-Form Grain Selector with a Varied Take-Off Angle	Single Crystal						Stray Grain		
	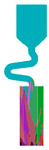	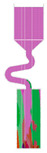	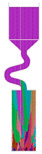	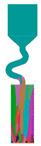	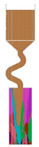	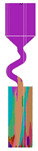	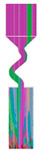	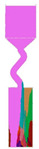	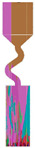
Stray Grain	No						Yes		
Probe	SZ *_t1_	SZ *_t2_	SZ *_t3_	SZ *_t4_	SZ *_t5_	SZ *_t6_	SZ *_t7_	SZ *_t8_	SZ *_t9_
Take-off angle	15°	20°	25°	30°	35°	40°	45°	50°	55°
Diameter (mm)	3								
Selector Height (mm)	10								
Starter Block Size (mm)	10 (L) × 10 (W) × 30 (H)								

* means the simulation serial number.
